# Crystal-Field Effects on Dipole Moments and Static First Hyperpolarizability in Noncentrosymmetric Ionic Organic Crystals

**DOI:** 10.1021/acsomega.6c05167

**Published:** 2026-07-16

**Authors:** Salviano A. Leão, Augusto César de Jesus, Marcos A. Castro, Tertius L. Fonseca

**Affiliations:** Instituto de Física, 67824 Universidade Federal de Goiás, Goiânia 74690-900, GO, Brazil

## Abstract

Understanding how crystal fields modify local nonlinear optical responses is essential for the rational design of acentric ionic organic crystals. Here, we investigate a structurally diverse set of noncentrosymmetric push−pull ionic crystals, including classical stilbazolium benchmarks and 6MNEP-related GUR/GUS crystals identified by their CCDC refcodes, to evaluate how electrostatic embedding affects the dipole moment and total static first hyperpolarizability (β_tot_) of asymmetric units. A self-consistent electrostatic-embedding approach combined with density functional theory (DFT) and time-dependent-DFT (TD-DFT) calculations was used to compare isolated and in-crystal ion-pair responses within a consistent local-descriptor framework. The results show that the crystal field does not act as a uniform amplifier or suppressor of β_tot_. Instead, its effect depends on the local electrostatic environment, ion-pair organization, and tensor-component balance. Classical stilbazolium salts generally retain large embedded hyperpolarizabilities, with DAPSH, DSTMS, DSCHS, and DSNS-1 among the most responsive systems. In contrast, most 6MNEP-related GUR/GUS crystals show substantial attenuation of β_tot_ upon embedding, while DSCHS, DSNS-1, and the structurally related MBST salt display enhanced embedded responses relative to their isolated counterparts. These results indicate that favorable local crystal-field alignment and contact patterns can reinforce, rather than suppress, the molecular response in selected cases. Overall, explicit crystal embedding provides a useful comparative strategy for analyzing how ion-pair composition and lattice organization modulate local dipolar and hyperpolarizability descriptors in ionic organic Organic nonlinear optical (NLO) crystals.

## Graphical abstract

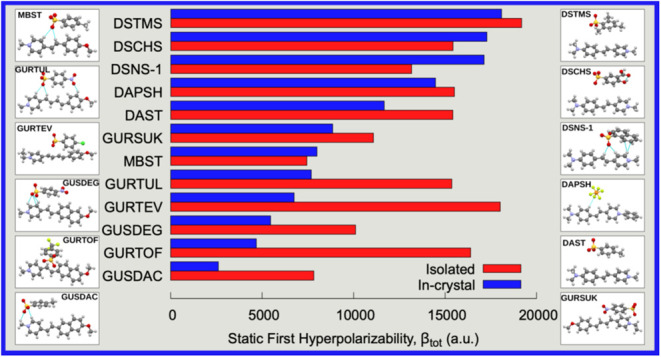

## 1 Introduction

Organic nonlinear optical (NLO) materials remain important in advanced photonics because they combine large second-order responses, broad optical transparency, and relatively mild processing conditions, enabling frequency conversion, terahertz (THz) generation/detection, and electro-optic modulation.^1–6^ Among them, organic salts built from push−pull chromophores (donor-π-acceptor, D-π-A) are particularly attractive because of their high molecular polarizability and strong coupling between electronic structure and external fields. However, a large molecular hyperpolarizability does not guarantee a strong solid-state response: the crystal must be noncentrosymmetric, and the orientational arrangement of the molecular tensors within the lattice must avoid extensive cancellation. Thus, the design of second-order NLO crystals requires consideration of both molecular electronic structure and the way the crystal environment perturbs and organizes that response in the solid state.

Recent experimental and theoretical studies have further reinforced the role of molecular and crystal engineering in controlling the optical response of functional molecular materials. Organic and organometallic NLO systems based on π-conjugation, donor−acceptor substitution, ion-pair organization, and crystal-environment control have shown that optical and nonlinear responses can be modulated by both molecular structure and supramolecular packing. For example, guanidinium benzenesulfonate and aminopyridine–*p*-aminobenzoic acid derivatives have been reported as organic crystals with tunable optical transparency, thermal stability, and second- or third-order NLO responses.^7,8^ Recent chalcone and stilbazolium derivatives further demonstrate how heterocyclic bridges, donor−acceptor substitution, halogen incorporation, and crystal growth conditions can affect SHG efficiency, optical limiting behavior, and photonic applicability.^9,10^ In parallel, theoretical studies on Pt­(II) complexes and recent reviews of push−pull chromophores emphasize that NLO responses are governed not only by intrinsic molecular hyperpolarizability but also by charge-transfer pathways, π-conjugation, substituent position, and the surrounding medium or crystal packing.^11,12^ Additional recent studies on thiourea derivatives, chiral organic−inorganic hybrid perovskites, amino-acid−based organic salts, halogenated organic crystals, mechanically bendable pyridine derivatives, ZnCl_2_-doped DAST crystals, and pyridinium-based ionic liquids further show that chemical substitution, chirality, hydrogen bonding, weak intermolecular contacts, dopant incorporation, and cation−anion coupling can substantially alter optical, electronic, and NLO behavior.^13–20^ These developments support the view that the design of efficient molecular NLO materials cannot rely only on highly polarizable chromophores but must also account for how the crystal environment and local ionic organization perturb dipolar and hyperpolarizability descriptors in the solid state.

A powerful way to tune the solid-state organization of D-π-A salts is counterion variation. Through electrostatic interactions, weak contacts (C−H···O/F), steric effects, and, in some cases, directional hydrogen bonds, the anion can influence how ion pairs are arranged in the crystal. This is illustrated by benchmark stilbazolium crystals such as DAST and DSTMS, which crystallize in noncentrosymmetric space groups and show strong SHG/THz performance, as well as by DAPSH, in which the weakly coordinating inorganic PF_6_^−^ anion is associated with a distinct packing regime.^2,21,22^ At the same time, because the present study treats the asymmetric unit as a complete ion pair, changes in the counterion should be viewed as affecting both supramolecular organization and the intrinsic electronic character of the embedded unit. Accordingly, comparisons among aryl sulfonates (tosylate, mesitylenesulfonate, nitro-/chloro-/trifluoromethyl-benzenesulfonates, 2-naphthalenesulfonate, sulfosalicylate) and PF_6_^−^ within related chromophore families provide a useful route to examine how ion-pair composition and crystal organization are associated with changes in local NLO descriptors.

Recent literature on stilbazolium-based organic ionic salts shows that the optical response of crystals arises from a balance between the intrinsic electronic structure of the ion pair and the perturbation imposed by the surrounding crystal environment.^23–30^ In push−pull systems with one-dimensional charge transfer, finite-field studies have shown that isotropic counterions may exert only a limited effect on the direction of the first molecular hyperpolarizability and a moderate effect on its magnitude, supporting cation-centered descriptions as a useful first approximation.^23^ Subsequent multiscale studies, however, demonstrated that this simplification is not always sufficient for interpreting crystal behavior, since the polarization field, the counterion, and supramolecular packing can substantially modify local molecular descriptors and their relation to bulk response.^24,25^ In parallel, analyses of intermolecular and interionic interactions in DAST derivatives indicated that changes in specific contacts can influence SHG performance,^26^ whereas integrated experimental-computational studies confirmed that the crystal environment modulates intramolecular charge transfer and reshapes the relative NLO potential of different stilbazolium salts.^27^ More recently, iterative/self-consistent electrostatic-embedding approaches combined with DFT/TD-DFT, applied to stilbazolium crystals, have shown that solid-state polarization can significantly affect dipole moments and molecular hyperpolarizabilities in the crystal environment and can improve agreement between local calculations and experimental trends.^28–30^ These studies collectively motivate an explicit treatment of crystal embedding when comparing ionic NLO materials.

In this work, we assemble and analyze a structurally diverse set of noncentrosymmetric ionic organic crystals. The set includes classical stilbazolium benchmarks, namely, DAST, DAPSH, DSTMS, DSCHS, and DSNS-1 reported in refs 2,21,22,31 , together with a group of 6MNEP-related push−pull pyridinium salts reported in ref 32 and identified here by their CCDC refcodes GURSUK, GURTEV, GURTOF, GURTUL, GUSDAC, and GUSDEG. MBST^33^ is also included as a structurally related methoxy-substituted stilbazolium-type salt. This collection does not constitute a single-variable series; instead, it provides a comparative framework for evaluating how ion-pair composition and crystal organization are associated with changes in local dipolar and hyperpolarizability descriptors under explicit crystal-field embedding. The CCDC refcodes and corresponding full standardized compound names, including common counterion descriptors, are provided in Table 1.

**Table 1:** Compounds Analyzed in This Work, Identified by CCDC Refcode, with Standardized Names Given in the “Cation–Anion” Format and Their Corresponding Counterions^a^

refcode	standardized compound name (cation–anion)	counterion (common name)
DAPSH CCDC 163560	4-[(E)-2-(4-(dimethylamino)phenyl)ethenyl]-1-phenylpyridin-1-ium hexafluorophosphate	PF_6_^−^ (hexafluorophosphate)
DAST CCDC 1175744	4-[4-(dimethylamino)styryl]-1-methylpyridin-1-ium 4-methylbenzenesulfonate	tosylate
DSCHS CCDC 781566	4-[4-(dimethylamino)styryl]-1-methylpyridin-1-ium 2-hydroxy-5-sulfobenzoate	sulfosalicylate
DSNS-1 CCDC 292763	4-[4-(dimethylamino)styryl]-1-methylpyridin-1-ium naphthalene-2-sulfonate	2-naphthalenesulfonate
DSTMS CCDC 277597	4-[4-(dimethylamino)styryl]-1-methylpyridin-1-ium 2,4,6-trimethylbenzenesulfonate	mesitylenesulfonate
GURTOF CCDC 1960849	1-ethyl-4-[(1E,3E)-4-(4-methoxyphenyl)buta-1,3-dien-1-yl]pyridin-1-ium 4-nitrobenzenesulfonate	*p*-nitrobenzenesulfonate
GURSUK CCDC 1960840	1-ethyl-4-[(1E,3E)-4-(4-methoxyphenyl)buta-1,3-dien-1-yl]pyridin-1-ium 4-chlorobenzenesulfonate	*p*-chlorobenzenesulfonate
GURTEV CCDC 1960847	1-methyl-4-[(1E,3E)-4-(4-methoxyphenyl)buta-1,3-dien-1-yl]pyridin-1-ium 4-(trifluoromethyl)benzenesulfonate	*p*-(trifluoromethyl)benzenesulfonate
GURTUL CCDC 1961452	1-methyl-4-[(1E,3E)-4-(4-methoxyphenyl)buta-1,3-dien-1-yl]pyridin-1-ium 4-nitrobenzenesulfonate	*p*-nitrobenzenesulfonate
GUSDAC CCDC 1867834	1-methyl-4-[(1E,3E)-4-(4-methoxyphenyl)buta-1,3-dien-1-yl]pyridin-1-ium 4-methylbenzenesulfonate	tosylate
GUSDEG CCDC 1867835	1-methyl-4-[(1E,3E)-4-(4-methoxyphenyl)buta-1,3-dien-1-yl]pyridin-1-ium 4-nitrobenzenesulfonate	*p*-nitrobenzenesulfonate
MBST CCDC 774844	1-methyl-4-[(E)-2-(4-methoxyphenyl)ethenyl]pyridin-1-ium 4-methylbenzenesulfonate	tosylate

a Note: The GUR/GUS labels correspond to CCDC refcodes of 6MNEP-related push−pull pyridinium salts reported in ref 32. They are used here only as crystallographic identifiers and do not denote an independent chemical series.

Our objective is to examine how the crystal environment modifies the dipole moment and static first hyperpolarizability of embedded asymmetric units in noncentrosymmetric D−π−A ionic crystals. In this context, crystallographic quantities such as lattice compactness and density are treated as descriptive variables of global packing compactness, whereas the calculated in-crystal properties are interpreted as local molecular descriptors within a consistent electrostatic-embedding scheme. Rather than assigning the response to a single crystallographic parameter, this study discusses how changes in counterion identity, local ion-pair arrangement, and lattice organization are associated with changes in embedded electronic response. The computational approach builds on previous self-consistent electrostatic-embedding methodologies that combine molecular quantum-mechanical (QM) calculations with an explicit representation of the surrounding crystal field.^34–36^ Related approaches have also been successfully used to investigate polarization effects in organic molecules in solution.^37–40^

## 2 Methodology and Computational Details

Quantum-chemical calculations based on density functional theory (DFT) were carried out using Gaussian 16.^41^ Exchange−correlation effects were described using a set of hybrid and long-range-corrected density functionals. Two long-range-corrected hybrid GGA functionals were used: LC-BLYP^42^ and CAM-B3LYP,^43^ in which the asymptotic behavior of the exchange interaction is improved by partitioning it into short- and long-range components. Two hybrid meta-GGA functionals, M05-2X^44^ and M06-2X,^45^ referred to as Minnesota functionals, were also employed; these functionals include an additional dependence on the local kinetic energy density. This set of functionals has previously been used to assess the applicability of DFT methods for calculating the hyperpolarizabilities of representative organic molecules.^46–49^ The 6-311+G­(d) basis set was used in all quantum-chemical calculations. The experimental crystal structures were used directly, without geometry optimization. Crystal-field effects on the asymmetric unit, taken here as the central quantum-chemical region, were described explicitly through the electrostatic field generated by the surrounding salts, represented by atomic point charges. A finite cluster containing 11 × 11 × 11 unit cells was employed, and all calculations were carried out without periodic boundary conditions. Molecular and crystal packing representations were generated with Mercury.^50^

The electrostatic embedding was determined self-consistently. The procedure started from CHELPG partial atomic charges^51^ fitted to the electrostatic potential generated by the CAM-B3LYP charge density of the isolated asymmetric unit and assigned to the surrounding equivalent units in the cluster. At each iteration, the central quantum-mechanical asymmetric unit was recalculated in the presence of the embedding field, new CHELPG charges were obtained, and the surrounding point charges were updated. The point charges belonging to the central quantum-mechanical region were excluded from the embedding field to avoid double counting. The procedure was repeated until both the total dipole moment (μ) and β_tot_ reached a stationary regime. Figure 1 shows representative convergence profiles of μ and β_tot_ for the benchmark crystals DAPSH, DAST, DSCHS, and DSTMS as a function of the iteration step in the self-consistent electrostatic-embedding procedure. The four systems illustrate the range of convergence behaviors observed in the present calculations, from rapid stabilization after the first iterations to more pronounced damped oscillations before reaching a stationary regime. These profiles confirm that the iterative embedding procedure leads to stable local dipolar and nonlinear optical descriptors for the embedded asymmetric units within the adopted finite-cluster model. In addition, the convergence of the calculated dipole moments for all noncentrosymmetric ionic crystals analyzed in this work is provided in Figure S1 of the Supporting Information. Recently, similar self-consistent electrostatic-embedding strategies have been applied to estimate linear and nonlinear optical properties in ionic and nonionic organic crystals.^52–58^

**Figure 1:**
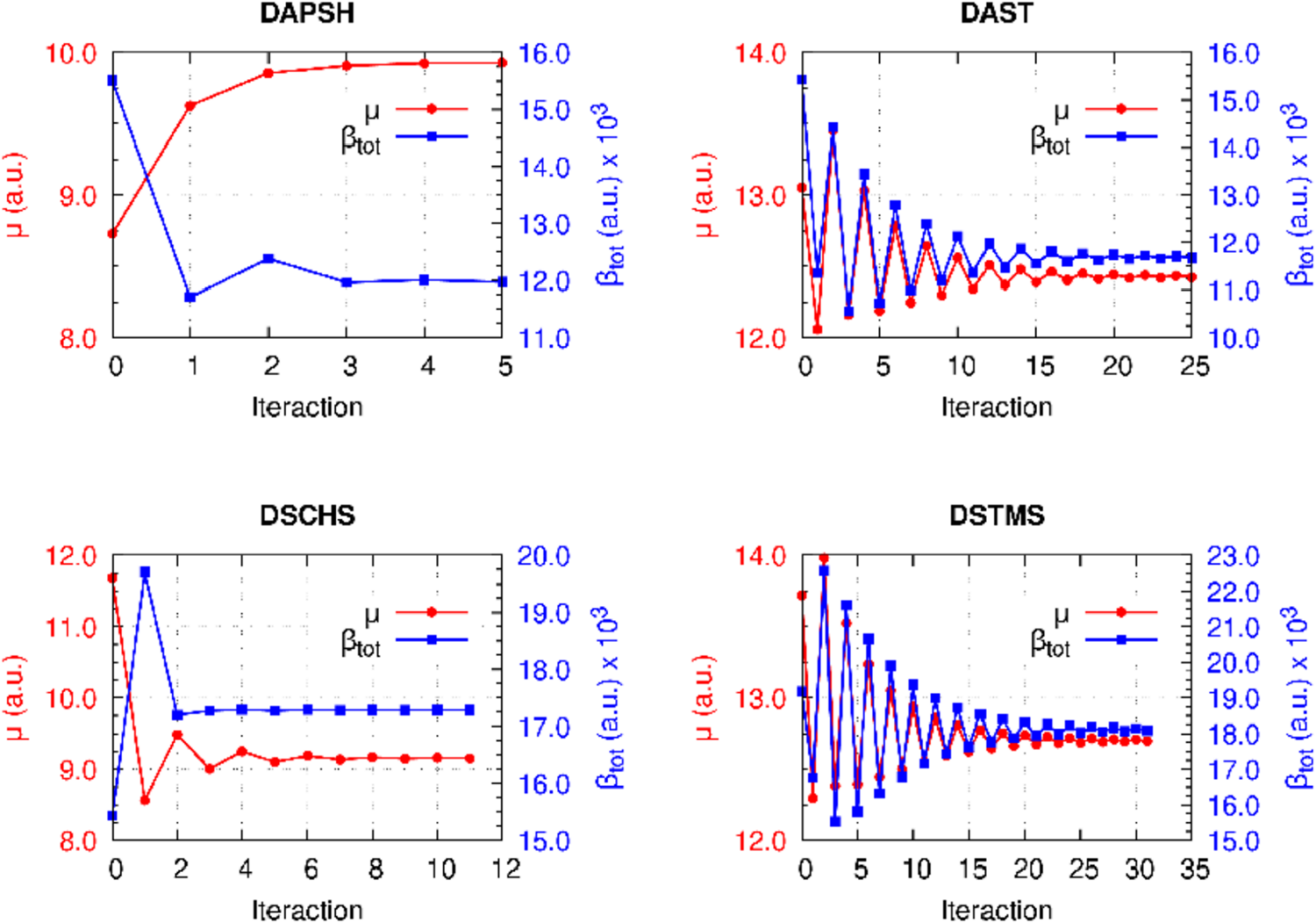
Convergence of the calculated dipole moment and total first hyperpolarizability of the asymmetric units of DAPSH, DAST, DSCHS, and DSTMS as a function of the iteration step in the self-consistent electrostatic-embedding procedure. The curves illustrate the stabilization of the local dipolar and nonlinear optical responses of the embedded asymmetric units within the adopted embedding model.

The present finite-cluster electrostatic-embedding model is complementary to periodic plane-wave approaches. Periodic methods are well suited to describe collective solid-state properties and fully periodic electronic structures, whereas the present strategy is designed to isolate the local response of a selected embedded ion pair under a self-consistent crystal electrostatic field. This framework enables a direct comparison between isolated and in-crystal asymmetric units using hybrid and long-range-corrected functionals, while preserving access to molecular descriptors such as ground- and excited-state dipole moments, transition dipole moments, tensor components of β_tot_, and two-level estimates. Therefore, the model is particularly useful for analyzing how local crystal fields perturb molecular NLO descriptors across chemically diverse ionic crystals. It should not be interpreted as a direct calculation of the macroscopic second-order susceptibility, but rather as a local comparative framework for assessing crystal-field effects on embedded molecular responses.^23,27^

The magnitude of the total first hyperpolarizability, β_tot_, is defined by eq 1,^59^ and the corresponding Cartesian components are given by eq 2. This convention allows the tensor response to be reduced to a scalar descriptor suitable for comparing the embedded asymmetric units across the crystal series.

1 $$\beta_{\mathrm{tot}}=\frac{1}{5}\sqrt{\beta_{x}^{2}+\beta_{y}^{2}+\beta_{z}^{2}}$$

and

2 $$\beta_{i}=\sum_{j}\left(\beta_{\mathrm{ijj}}+\beta_{\mathrm{jji}}+\beta_{\mathrm{jij}}\right)i,j=\left(x,y,z\right)$$

Excited-state quantities relevant to the present analysis, including vertical excitation energies, transition dipole moments, and ground-to-excited-state dipole-moment changes, were computed using time-dependent density functional theory (TD-DFT).^60–62^ These calculations were carried out with the same set of long-range-corrected hybrid GGA and hybrid meta-GGA functionals used for the ground-state analysis, allowing a consistent comparison of trends across the series, particularly for systems in which charge-transfer contributions are important.^63^

## 3 Results and Discussion

### 3.1 Crystal Structure Analysis

The salts analyzed here, whose single-crystal X-ray diffraction data have been reported previously,^2,21,22,31–33^ crystallize in noncentrosymmetric space groups (*P*2_1_2_1_2_1_, *Pn*, *P*1, or *Cc*), thereby fulfilling the crystallographic prerequisite for second-order optical activity. This observation, however, should be regarded only as a structural condition and not as a predictor of response magnitude, since the latter also depends on the orientational distribution of the molecular tensors within the crystal and on the interaction of each ion pair with its surroundings. The calculations were based on previously reported experimental single-crystal structures deposited in the CCDC database, whose refcodes are listed in Table 1. Additional crystallographic parameters, including space group, unit-cell dimensions, unit-cell volume, number of asymmetric units per unit cell (*Z*), volume per asymmetric unit (V/Z), and density (ρ), are summarized in Table S1 of the Supporting Information. Figure 2 presents the chemical structures of the asymmetric units of the noncentrosymmetric organic salts analyzed in this work.

**Figure 2:**
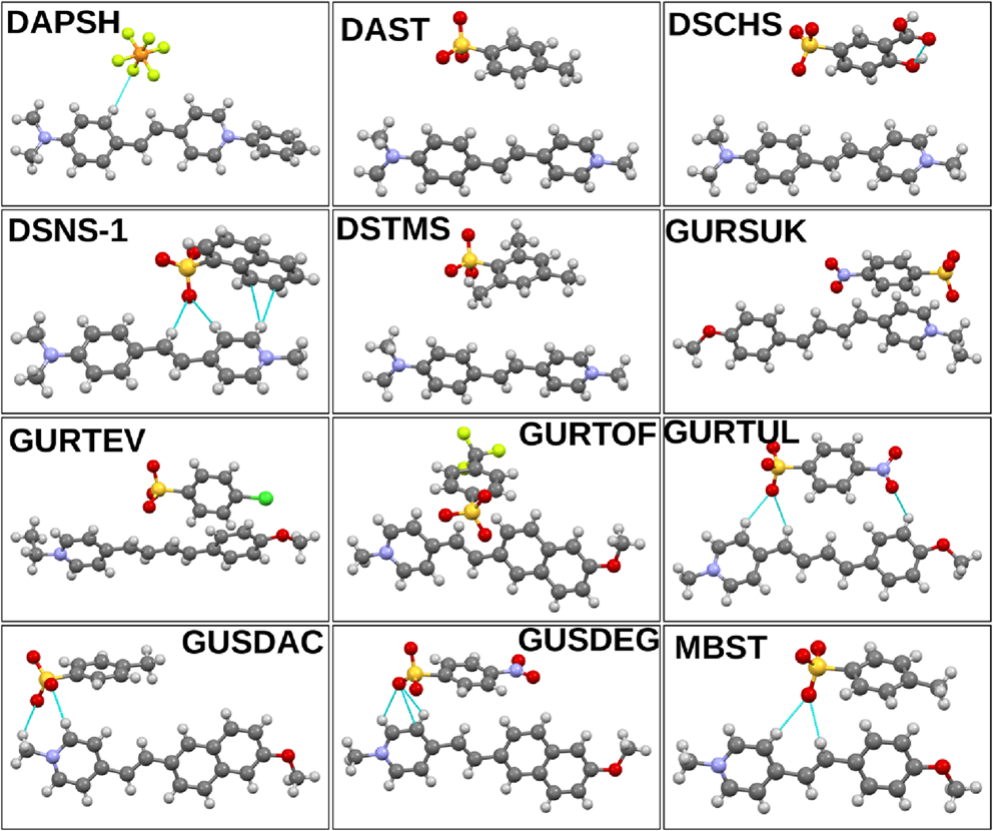
Chemical structures of the asymmetric units of the noncentrosymmetric ionic crystals analyzed in this work, using a consistent crystallographic definition of the asymmetric unit across all crystals. The representations were generated with Mercury.^50^

Within the 6MNEP-related GUR subset, GURSUK, GURTEV, and GURTUL form a closely related orthorhombic set, all crystallizing in *P*2_1_2_1_2_1_ with very similar unit-cell metrics. These labels correspond to CCDC refcodes of 6MNEP-related push−pull pyridinium salts rather than to an independent chemical series. This isostructural relationship indicates preservation of a common packing motif across these three structures and provides a useful basis for comparing how counterion substitution perturbs an otherwise conserved crystallographic framework. GURTOF, in contrast, crystallizes in monoclinic Pn and therefore represents a distinct packing arrangement within the same chromophore family. In structural terms, the GUR subset thus contains both a conserved packing platform and a clear outlier associated with broader lattice reorganization.

A second regime is formed by the 6MNEP-related GUS crystals, GUSDAC and GUSDEG, which crystallize in triclinic P1. MBST also crystallizes in triclinic P1 and is structurally related as a methoxy-substituted stilbazolium-type salt, but it is not treated here as part of the same 6MNEP-derived GUR/GUS group. The lower symmetry of this triclinic subgroup allows a wider variety of relative ion-pair orientations and intermolecular-contact patterns than in the orthorhombic GUR platform. At the crystallographic level, the main result is therefore the identification of a distinct low-symmetry packing regime rather than a direct inference about the nonlinear optical efficiency.

The benchmark stilbazolium salts DAST, DSTMS, and DAPSH crystallize in monoclinic *Cc* and provide a useful reference group for comparing structurally related cation−anion assemblies within a common symmetry class. Even within this shared space-group setting, the structures display distinct packing efficiencies and contact networks, especially when the arylsulfonate salts are compared with DAPSH, which contains the PF_6_^−^ anion, with an even weaker coordination capacity. Because the asymmetric unit employed in the subsequent calculations is the complete ion pair, these counterion-dependent differences should be interpreted as combined changes in ion-pair identity and crystal packing, rather than as purely packing-mediated effects.

Additional structural diversity is introduced by DSCHS and DSNS-1, both of which crystallize in triclinic P1. Among them, DSCHS is particularly distinctive because the sulfosalicylate anion supports classical O−H···O hydrogen-bonding motifs, giving rise to a more directional supramolecular network than that expected for sulfonate salts lacking an acidic hydroxyl group. In the present section, such interactions are treated as experimentally established structural features that help characterize the packing motif; no direct quantitative relationship between these contacts and the magnitude of the nonlinear response is assumed at this stage.

Overall, the crystallographic data define a set of clearly differentiated packing situations: an isostructural orthorhombic 6MNEP-related GUR platform (GURSUK/GURTEV/GURTUL), a monoclinic 6MNEP-related GURTOF outlier, a triclinic low-symmetry group, including the 6MNEP-related GUS crystals and the structurally related MBST, DSCHS, and DSNS-1 salts, and a monoclinic benchmark stilbazolium group (DAST/DSTMS/DAPSH). This classification provides a structural basis for the subsequent comparison of the embedded electronic properties. At the same time, it is important to emphasize that the crystal structure analysis is descriptive: it identifies differences in symmetry, packing families, and contact motifs, but it does not by itself quantify local field anisotropy or predict the magnitude of the in-crystal first hyperpolarizability.

### 3.2 Dipole Moments and Ground-to-Excited-State Dipole Contrast

To assess how the crystal environment modifies the electronic polarization of the ionic chromophores, we analyzed the ground-state dipole moments (μ_g_), excited-state dipole moments (μ_e_), and the ground-to-excited-state dipole-moment variation (Δμ). Within the present embedding framework, these quantities refer to the asymmetric units adopted as the central QM regions and are therefore used as local comparative descriptors of the embedded ion pairs rather than as unique observables of the crystal as a whole. Although their absolute values can depend on the chosen local partition of the crystal, all comparisons below use the same crystallographic definition of the asymmetric unit.^50^ Because Δμ is sensitive to charge redistribution upon excitation, these quantities provide a useful measure of how the local electrostatic environment perturbs the electronic structure beyond global crystallographic descriptors such as V/Z and ρ. Importantly, Δμ is evaluated here as the norm of the vector difference between the excited- and ground-state dipole moments, Δμ = |μ⃗_e_ − μ⃗_g_|, and not as the difference between the scalar dipole-moment magnitudes. Thus, systems with similar μ_g_ and μ_e_ magnitudes may still exhibit a non-negligible Δμ when the corresponding dipole vectors are not collinear. The exchange-correlation effects on μ_g_ and μ_e_ of the in-crystal asymmetric units were further assessed with CAM-B3LYP, LC-BLYP, M05-2X, and M06-2X using the 6-311+G­(d) basis set, as summarized in Table S2 of the Supporting Information. The ground-state dipole moments are consistently reproduced across the four functionals, indicating that the embedding-induced ground-state polarization is only weakly dependent on the exchange-correlation treatment. The excited-state dipole moments show somewhat larger functional sensitivity in selected cases, but the main qualitative trends across the series are retained. Accordingly, the discussion below focuses on the CAM-B3LYP results as a representative reference, while keeping in mind that excited-state dipolar properties are more method-dependent than the corresponding ground-state values.

CAM-B3LYP/6-311+G­(d) dipolar properties for the isolated and in-crystal asymmetric units are collected in Table 2. The isolated asymmetric units already suggest two broad patterns of dipolar response. The stilbazolium-based salts, including DAPSH, DAST, DSCHS, DSNS-1, and DSTMS, display moderate-to-large ground-state dipole moments and excited-state dipoles of comparable magnitude, leading to moderate Δμ values. In contrast, the 6MNEP-related GUR/GUS crystals and the structurally related MBST salt are characterized, in the isolated state, by excited-state dipole moment magnitudes that are generally smaller than their ground-state counterparts, which results in larger vectorial Δμ values. These differences indicate that these compounds undergo distinct degrees of dipolar rearrangement upon excitation already at the isolated ion-pair level, before inclusion of the crystal environment.

**Table 2:** CAM-B3LYP/6-311+G­(d) Results for the Ground-State Dipole Moment (μ_g_, in a.u.), Excited-State Dipole Moment (μ_e_, in a.u.), and Ground-to-Excited-State Dipole-Moment Variation, Defined as the Norm of the Vector Difference Δμ = |μ⃗_e_ − μ⃗_g_|, for the Isolated and In-Crystal Asymmetric Units of the Ionic Crystals

	isolated			in-crystal		
asymmetric unit	μ_g_	μ_e_	Δμ	μ_g_	μ_e_	Δμ
DAPSH	8.727	9.458	3.061	9.929	10.124	3.183
DAST	13.053	11.722	2.661	12.423	12.441	0.751
DSCHS	11.682	10.691	3.211	9.155	8.318	1.998
DSNS-1	8.008	7.875	4.958	7.731	7.875	4.486
DSTMS	13.721	10.738	4.362	12.699	12.685	0.693
GURSUK	7.720	2.025	7.903	8.913	11.628	3.973
GURTEV	8.964	1.843	7.953	11.232	9.146	3.410
GURTOF	13.257	2.743	11.786	15.879	13.681	3.160
GURTUL	9.883	2.497	8.501	12.021	12.422	3.107
GUSDAC	8.298	3.672	8.399	7.317	8.108	2.057
GUSDEG	8.609	2.322	9.127	7.610	4.328	3.513
MBST	8.930	0.991	8.610	7.661	7.769	3.514

Embedding the chromophores in the crystal environment shows that the dipolar descriptors of the classical stilbazolium salts are modified to different extents depending on the salt. For DAST and DSTMS, Δμ decreases substantially in the crystal, reflecting a marked reduction in the contrast between the ground- and excited-state dipole vectors within the embedded ion pair. DSCHS shows the same general tendency, although less strongly. By contrast, DAPSH retains a nearly unchanged Δμ upon crystallization, while DSNS-1 preserves a comparatively large dipole-moment change in the embedded state. These trends are accompanied by distinct changes in μ_g_ and μ_e_: in DAST and DSTMS, the in-crystal excited-state dipole becomes much closer in magnitude to the ground-state value, whereas in DAPSH and DSNS-1, the two dipolar states remain more clearly differentiated. The embedded values therefore reveal that the crystal environment does not affect this subset uniformly; instead, the magnitude and direction of the dipolar perturbation remain salt-dependent even within the same broader stilbazolium class. It is noted that the μ_g_ value obtained for DAPSH differs from the value reported in ref 29 because a different reference ion pair within the unit cell was selected for the local asymmetric unit calculation: here, we define the first ion pair such that PF_6_^−^ is located at (*x* + 1/2, *y*, *z* + 1/2) relative to the coordinates in the crystallographic information file (CIF). This alternative unit-cell origin/ion-pair definition changes the assigned molecular dipole without affecting the underlying crystal structure.

A comparison with the finite-field study of Kim et al.^23^ further highlights the sensitivity of dipole moment values to the molecular fragment and electrostatic model used to represent the ionic crystal environment. For DAPSH, Kim et al.^23^ reported a very small dipole moment for the isolated cation, μ_total_ = 0.6 au, whereas the inclusion of neighboring anionic species led to larger values, depending on how the counteranions were represented. When the neighboring PF_6_^−^ anions were modeled as point charges located at the phosphorus positions, the dipole moment increased to μ_total_ = 1.8 au when all surrounding point charges were included. When the PF_6_^−^ anions were treated explicitly, the corresponding value increased to μ_total_ = 5.0 au. These results show that the calculated dipole moment is strongly affected by the electrostatic representation of the counteranion environment. In the present work, DAPSH is described as a complete ion pair already in the isolated asymmetric unit calculation, giving μ_g_ = 8.727 au, and is then embedded in a self-consistent crystal electrostatic field, leading to μ_g_ = 9.929 au. This difference is expected because the present descriptor refers to the full asymmetric unit and its embedded response, rather than to the isolated cation or to a cation perturbed by a finite first-neighbor anionic environment. Therefore, the comparison with Kim et al. should not be interpreted as a direct numerical validation of the absolute dipole moments but rather as supporting the same qualitative conclusion: the counteranion and the crystal electrostatic environment substantially modulate the local dipolar response of ionic NLO chromophores.

A different overall response is observed for the 6MNEP-related GUR/GUS crystals and the structurally related MBST salt. In the GUR/GUS group, embedding generally increases the excited-state dipole moment relative to the isolated value, while the final Δμ values become concentrated within a narrower range than in the isolated species. This behavior indicates that the excited-state dipolar descriptors of these salts are particularly sensitive to the crystal environment. Rather than preserving the isolated-state pattern, the embedding field substantially reshapes the balance between ground- and excited-state polarization across the group. MBST also shows a strong embedding-induced change in the dipolar response. Although its embedded μ_g_ and μ_e_ magnitudes are very similar, the corresponding dipole vectors are not collinear, resulting in a non-negligible vectorial Δμ value. As a result, the broad dispersion of isolated Δμ values is replaced by a more compressed distribution in the embedded calculations, showing that the crystal field tends to attenuate the initially large dipolar contrast of these ion pairs.

The in-crystal versus isolated Δμ plot further summarizes this behavior (Figure 3). Points below the line indicate the attenuation of Δμ upon crystal embedding, whereas points above the line indicate enhancement. Except for DAPSH, all systems lie on or below this line, showing that crystal embedding generally reduces the ground-to-excited-state dipole-moment variation relative to the isolated asymmetric unit. This attenuation is particularly pronounced for the 6MNEP-related GUR/GUS crystals, whereas DAPSH and DSNS-1 retain much of their isolated dipolar contrast. MBST also remains below the parity line, confirming that embedding reduces its initially large isolated Δμ. Overall, the crystal environment reduces Δμ and compresses its distribution across the series, although the magnitude of this effect remains compound-dependent. Because Δμ includes changes in both the magnitude and direction of the dipole moment upon excitation, the isolated Δμ value alone is not a reliable predictor of the corresponding in-crystal dipolar response.

**Figure 3:**
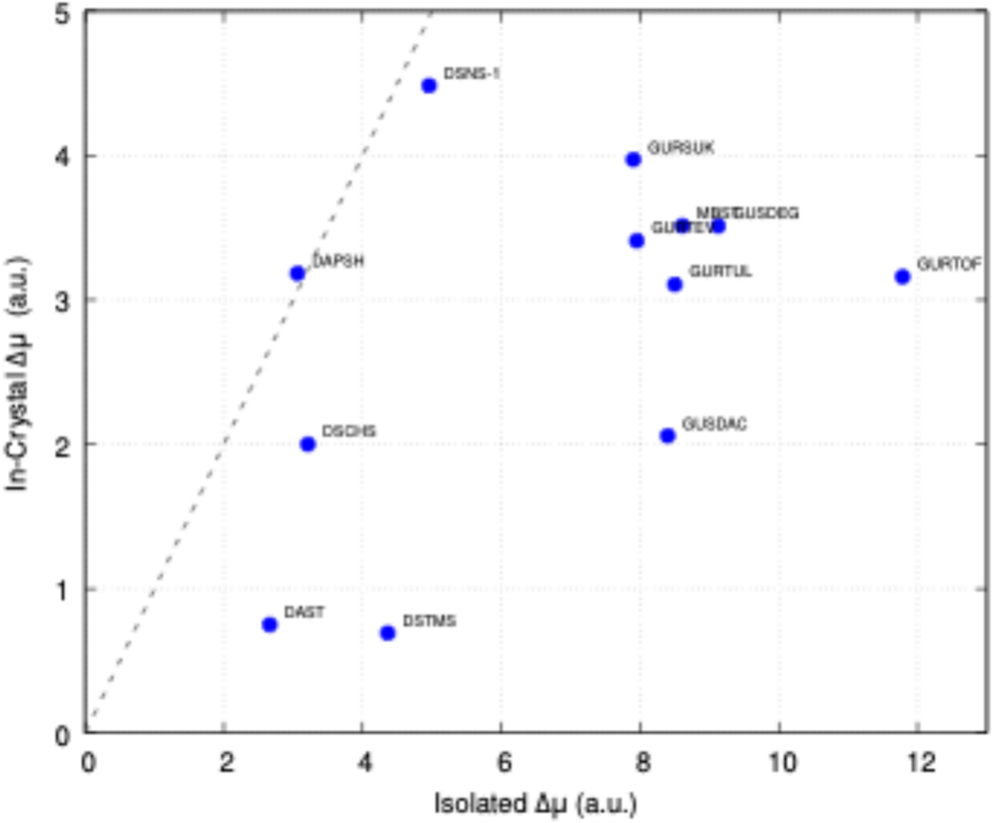
Correlation between isolated and in-crystal ground-to-excited-state dipole-moment variations, Δμ = |μ⃗_e_ − μ⃗_g_|, for the asymmetric units. The dashed line (in-crystal Δμ = isolated Δμ) marks parity between the two conditions.

Taken together, these results show that crystallization can influence the dipolar response in more than one way: it may reduce the ground-to-excited-state dipole contrast, as observed for DAST and DSTMS, or it may substantially reorganize the excited-state dipolar pattern, as seen for the 6MNEP-related GUR/GUS crystals and MBST. More broadly, μ_g_, μ_e_, and the vectorial descriptor Δμ provide a compact electronic description of how the embedded asymmetric units respond to their crystal environment and help contextualize the trends discussed later for the first hyperpolarizability.

### 3.3 Crystal Field Effects on Static First Hyperpolarizability

The dipolar trends discussed above indicate that crystal embedding can either preserve or substantially reorganize the local electronic response of the asymmetric units, particularly through changes in the balance between ground- and excited-state polarization. Because such changes are directly relevant to second-order nonlinear optical behavior, we next examine how the same crystal environment affects the total static first hyperpolarizability (β_tot_). The in-crystal β_tot_ values calculated with CAM-B3LYP, LC-BLYP, M05-2X, and M06-2X using the 6-311+G­(d) basis set show broad qualitative consistency across the series, although the absolute magnitudes remain noticeably dependent on the exchange-correlation functional (Table 3). For several salts, the relative grouping of higher- and lower-β_tot_ systems is preserved across the four functionals, but quantitative differences are not negligible and become larger for some of the most responsive compounds. In many instances, CAM-B3LYP, M05-2X, and M06-2X yield comparable values, whereas LC-BLYP shows a less uniform behavior, producing either larger or smaller responses depending on the compound. These results indicate that the calculated hyperpolarizabilities are best interpreted as comparative trends within a consistent electronic-structure framework rather than as numerically definitive predictions.

**Table 3:** DFT/6-311+G­(d) Results for the Static First Hyperpolarizability (β_tot_, in a.u.) of In-Crystal Asymmetric Units of Ionic Crystals

asymmetric unit	CAM-B3LYP	LC-BLYP	M05-2X	M06-2X
DAPSH	14,475.08	14,077.61	14,322.65	15,280.39
DAST	11,672.54	16,878.26	10,445.47	10,229.43
DSCHS	17,283.27	21,584.40	16,598.03	16,954.76
DSNS-1	17,130.81	12,148.43	16,922.52	18,179.14
DSTMS	18,086.31	32,428.04	16,347.10	16,098.90
GURSUK	8855.24	6699.83	9027.72	9390.41
GURTEV	6748.82	5063.35	6751.17	6969.96
GURTOF	4683.67	3598.35	4561.52	4770.66
GURTUL	7685.80	5722.01	7510.45	8006.67
GUSDAC	2606.22	2093.63	2552.51	2671.53
GUSDEG	5462.35	4129.61	5368.30	5706.05
MBST	7996.92	6599.14	7597.16	8168.67

Among the tested functionals, CAM-B3LYP was adopted as the reference for the discussion below because it provides a balanced description of long-range charge-transfer effects and is used consistently throughout the electrostatic-embedding procedure. This choice should be regarded as a practical reference rather than as a unique validation for every salt in the set. In addition, the β_tot_ values discussed here correspond to embedded asymmetric units within the adopted partitioning of the crystal and therefore represent local molecular descriptors in the crystal environment.

When examined alongside the structural groupings identified above, the CAM-B3LYP/6-311+G­(d) results in Table 4 show that the isolated first hyperpolarizabilities span a substantial range, from approximately 7.44 × 10^3^ to 1.92 × 10^4^ a.u. The highest isolated responses are found for DSTMS (19182 au), GURTEV (18013 au), and GURTOF (16396 au), while DAPSH, DAST, DSCHS, and GURTUL form an intermediate-high group clustered around 1.5 × 10^4^ a.u. DSNS-1, GURSUK, and GUSDEG occupy an intermediate range, whereas GUSDAC and MBST display the lowest isolated values in the series. Because these quantities are calculated for isolated ion pairs, they primarily reflect the intrinsic electronic asymmetry and charge-transfer characteristics of the selected asymmetric units, rather than crystal packing itself. In this sense, global crystallographic descriptors such as V/Z and ρ are not expected to determine the isolated β_tot_ values.

**Table 4:** CAM-B3LYP/6-311+G­(d) Results for Static First Hyperpolarizability (β_tot_, in a.u.) of Isolated and In-crystal Asymmetric Units

	β_tot_	
asymmetric unit	isolated	in-crystal
DAPSH	15,508.95	14,475.08
DAST	15,423.58	11,672.54
DSCHS	15,433.38	17,283.27
DSNS-1	13,170.86	17,130.81
DSTMS	19,182.02	18,086.31
GURSUK	11,083.98	8855.24
GURTEV	18,013.21	6748.82
GURTOF	16,396.02	4683.67
GURTUL	15,367.41	7685.80
GUSDAC	7824.93	2606.22
GUSDEG	10,106.35	5462.35
MBST	7444.32	7996.92

Embedding the asymmetric units in the crystal environment changes this picture appreciably. At the CAM-B3LYP level, the in-crystal β_tot_ values span from 2.61 × 10^3^ to 1.81 × 10^4^ a.u., showing that the electrostatic environment represented in the present electrostatic-embedding model can either preserve, attenuate, or enhance the local molecular response. DSCHS and DSNS-1 display clear increases relative to their isolated values, while DSTMS remains close to the isolated limit. DAPSH and DAST show moderate reductions upon embedding. In contrast, most 6MNEP-related GUR/GUS crystals undergo substantial attenuation, with particularly strong decreases for GURTEV, GURTOF, GURTUL, and GUSDAC. MBST, although structurally related to this broader set of methoxy-substituted push−pull salts, behaves as a distinct case, showing a slight increase in the embedded calculation. Taken together, these comparisons indicate that the local crystal environment can strongly influence the calculated β_tot_ of the embedded ion pair, although the present data do not by themselves identify a single structural descriptor as uniquely responsible for the observed changes.

Figure 4 provides a compact summary of this comparison by correlating the in-crystal and isolated first hyperpolarizabilities of the asymmetric units. The dashed in-crystal β_tot_ = isolated β_tot_ line separates enhancement from attenuation within the present embedding scheme. DSCHS and DSNS-1 lie clearly above the diagonal, and MBST appears slightly above it, whereas DSTMS and DAPSH remain close to parity but on the attenuation side. DAST, GURSUK, GURTEV, GURTOF, GURTUL, GUSDAC, and GUSDEG fall more clearly below the line. The resulting spread around the parity condition shows that the isolated β_tot_ of the ion pair is not, by itself, sufficient to anticipate the corresponding embedded value. Instead, the calculated response depends on how the electronic structure of the asymmetric unit is perturbed by the surrounding lattice within the adopted electrostatic model. This comparison is therefore useful for identifying environment-sensitive systems and may provide a qualitative indication of how strongly the local molecular response is affected by the crystal environment.

**Figure 4:**
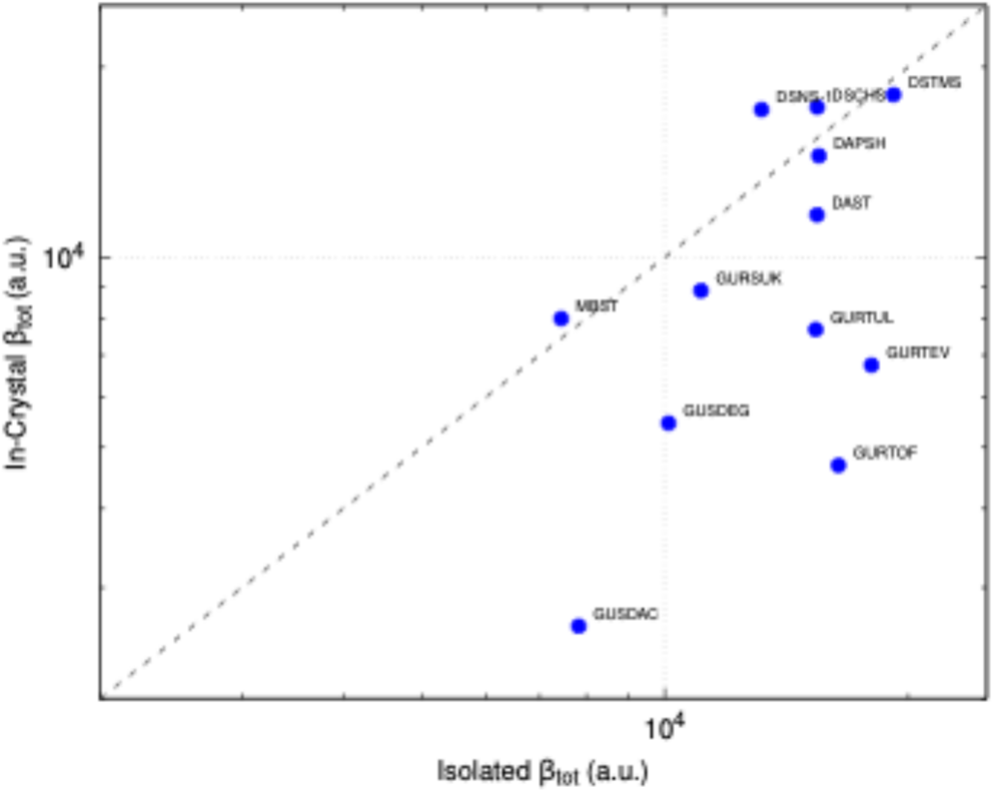
Correlation between isolated and in-crystal first hyperpolarizabilities for the asymmetric units. The dashed line (in-crystal β_tot_ = isolated β_tot_) separates enhancement from attenuation within the present electrostatic-embedding model.

The enhancement observed for DSCHS, DSNS-1, and MBST deserves particular attention. These salts show larger β_tot_ values in the crystal than in the isolated ion pair, indicating that, in selected cases, the local electrostatic environment can reinforce rather than attenuate the molecular response. However, this behavior does not appear to be controlled by a single global packing descriptor, such as V/Z, density, or space group. Instead, it is more plausibly associated with compound-specific local factors, including ion-pair organization, the directionality of anion−cation contacts, hydrogen-bonding or short-contact motifs, and the alignment between the crystal field and the molecular charge-transfer axis. DSCHS is especially relevant in this context because the sulfosalicylate anion can support directional O−H···O interactions, whereas DSNS-1 and MBST illustrate that β_tot_ enhancement can also arise in other low-symmetry packing environments where short-contact directionality and local electrostatic alignment may be favorable. Thus, the increase in β_tot_ for these systems should be interpreted as a local crystal-field effect rather than as a consequence of global compactness or crystallographic symmetry alone.

It is also important to emphasize that the embedded β_tot_ trends do not follow the dipole-moment variation in a simple one-to-one manner. As discussed above, Δμ is a vectorial descriptor that reflects changes in both magnitude and direction of the dipole moment upon excitation. Nevertheless, the total static first hyperpolarizability depends on the full tensor response, including excitation energies, transition dipole moments, multistate contributions, and possible cancellation or reinforcement among tensor components. MBST provides a useful example: although its embedded μ_g_ and μ_e_ magnitudes are similar, the corresponding dipole vectors are not collinear, so the vectorial Δμ remains non-negligible. At the same time, its β_tot_ is slightly enhanced in the crystal. This behavior reinforces that Δμ is useful for interpreting charge redistribution, but it is not sufficient by itself to predict the full embedded β_tot_ response.

### 3.4 First Hyperpolarizability within the Two-Level Model (β_tot_^TL^)

The four functionals provide a broadly similar two-level picture, in which larger β_tot_^TL^ values are generally associated, within the standard two-level expression ( $\beta_{\mathrm{tot}}^{\mathrm{TL}}\propto \left(\mu_{\mathrm{ge}}^{2}\Delta \mu /E_{\mathrm{ge}}^{2}\right)\sqrt{1+8\cos{\;}^{2}\theta }$ ),^59^ with lower excitation energies (*E*_ge_), larger transition dipole moments (μ_tr_), sizable ground-to-excited-state dipole-moment changes, and ground- and excited-state dipole vectors that are nearly collinear (*cos* θ close to ± 1) (Tables S3−S6, Supporting Information). At the same time, the quantitative values remain functional-dependent, particularly in cases where Δμ or the angular term varies appreciably. Across the present series, DAPSH and DSNS-1 remain among the most favored systems in most functionals, with DSCHS and GURSUK also displaying consistently large β_tot_^TL^ values. DAST shows only moderate two-level response despite its large μ_tr_, because its comparatively small Δμ limits the final estimate. DSTMS follows the same general pattern in CAM-B3LYP, M05-2X, and M06-2X but becomes a clear exception at the LC-BLYP level, where a larger Δμ, combined with large μ_tr_ and near-collinear dipole vectors, leads to a much stronger β_tot_^TL^. The 6MNEP-related GUR/GUS crystals occupy an intermediate region overall, with GURSUK remaining more consistently responsive than GURTEV, GURTUL, and GURTOF. GUSDAC remains weak across all functionals because of its limited Δμ, whereas GUSDEG is particularly method-sensitive, showing that the two-level estimate can vary substantially when the relative orientation of the ground- and excited-state dipoles changes. MBST behaves as a structurally related but distinct case, with β_tot_^TL^ values that remain relatively high across the four functionals, reflecting the combined influence of μ_tr_, vectorial Δμ, and dipole alignment. Overall, the four functionals preserve the main qualitative structure−property relationships, while the quantitative differences in β_tot_^TL^ can be traced primarily to functional-dependent changes in *E*_ge_, μ_tr_, Δμ, and, in selected cases, the angular factor.

Comparison of the DFT static first hyperpolarizabilities (β_tot_, Table 3) with the corresponding two-level estimates (β_tot_^TL^, Table 5) shows that the two-level model captures part of the qualitative hierarchy of the series, but not its full quantitative response in all cases. In general, salts identified as highly responsive from the full DFT calculations—particularly DAPSH, DSCHS, DSNS-1, and, depending on the functional, DSTMS—also display large β_tot_^TL^ values, indicating that the dominant low-energy transition provides a useful first-order interpretation of the embedded molecular response. The agreement is most satisfactory for systems such as DAPSH and DSNS-1, for which β_tot_ and β_tot_^TL^ remain of the same order of magnitude across the four functionals, and remains reasonable for DSCHS. At the same time, important discrepancies define the limits of the approximation. DSTMS is a representative example: its DFT β_tot_ remains large in all functionals, whereas β_tot_^TL^ is comparatively modest in CAM-B3LYP, M05-2X, and M06-2X and becomes very large only at the LC-BLYP level, showing that the full static response cannot always be reduced to a single dominant excitation. A complementary behavior is found for several 6MNEP-related GUR/GUS crystals and for MBST, for which β_tot_^TL^ often exceeds the corresponding DFT β_tot_. This suggests that although the leading transition may be electronically favorable within the two-level picture, the final static response can be reduced by effects that are not captured by the single-transition approximation, such as multistate contributions and tensor-component cancellation. Overall, β_tot_^TL^ is best regarded as a mechanistic and semiquantitative descriptor: it is useful for identifying how low *E*_ge_, large μ_tr_, significant Δμ, and favorable dipole alignment can contribute to enhanced response, whereas the full DFT β_tot_ remains necessary to describe the complete in-crystal first hyperpolarizability of the embedded asymmetric units.

**Table 5:** DFT Results for the In-crystal First Hyperpolarizability Estimated from the Two-Level Model (β_tot_^TL^, in a.u.) for the Asymmetric Units

asymmetric unit	CAM-B3LYP	LC-BLYP	M05-2X	M06-2X
DAPSH	17,495.09	18,083.13	19,155.50	19,798.38
DAST	8127.56	13,718.60	7123.20	6753.56
DSCHS	15,695.03	24,572.79	13,480.11	13,536.69
DSNS-1	15,738.55	16,768.92	21,335.17	22,457.64
DSTMS	8953.30	29,029.81	7583.96	6931.86
GURSUK	15,220.56	11,110.78	15,386.84	16,257.19
GURTEV	12,218.90	8380.75	12,296.46	12,876.26
GURTOF	8420.32	5525.56	8254.62	8958.01
GURTUL	8914.13	9096.61	11,144.96	13,373.86
GUSDAC	4393.80	3175.62	4391.28	5280.90
GUSDEG	2164.11	6792.52	10,003.76	10,428.53
MBST	10,525.20	8692.57	10,108.49	10,804.24

### 3.5 Comparison with Previous Hyperpolarizability Studies

The static first hyperpolarizability results obtained in the present study are consistent with previous investigations showing that the nonlinear optical response of stilbazolium-based ionic crystals is strongly affected by the crystal environment. However, the magnitude and even the direction of this effect may depend on the hyperpolarizability descriptor considered, the molecular fragment used for analysis, and the way in which the solid-state environment is represented.

Kim et al.^23^ investigated DAPSH using finite-field calculations and reported β_max_ = 208.3 × 10^−30^ esu for the isolated cation and β_max_ = 244.9 × 10^−30^ esu when neighboring PF_6_^−^ counteranions were included. In the present calculations, DAPSH shows a different but complementary behavior: the CAM-B3LYP/6-311+G­(d) β_tot_ value decreases slightly from 15,508.95 au for the isolated asymmetric unit to 14475.08 au in the embedded crystal environment, corresponding to approximately 134.0 and 125.1 × 10^−30^ esu, respectively. This apparent difference is expected because Kim et al. analyzed β_max_ along the main charge-transfer direction of the cation, whereas the present work evaluates β_*tot*_ for the complete asymmetric unit under a self-consistent electrostatic-embedding field. Therefore, both studies support the same mechanistic view that counterions and the crystal electrostatic environment actively modulate the first hyperpolarizability of ionic NLO chromophores.

A useful complementary comparison can be made with the work of Cole et al.,^26^ who analyzed DAST and structurally related stilbazolium derivatives by separating molecular, crystallographic, and intermolecular contributions to second-order nonlinear optical response. In that study, the authors introduced the parameter η_inter_ to isolate the effect of intermolecular crystal-field forces on SHG efficiency after accounting for molecular hyperpolarizability, molecular number density, and chromophore orientation within the crystal. Their results showed that the NLO response of DAST-type ionic crystals is not governed only by the intrinsic β of the chromophore or by simple global packing descriptors. Instead, specific local cation−anion interactions, especially the concerted contribution of H···O/F and H···C­(π) contacts, were found to play a decisive role in modulating the effective nonlinear response.

This interpretation is directly relevant to the present results. In our embedded ion-pair calculations, the crystal field also does not act as a uniform amplifier or suppressor of β_tot_. Rather, its effect depends on the local electrostatic environment, the relative organization of cations and anions, and the alignment between the local crystal field and the molecular charge-transfer axis. Thus, the comparison with Cole et al. supports the view that local supramolecular organization must be considered explicitly when rationalizing the nonlinear optical response of ionic organic crystals. While their analysis correlated SHG performance with experimentally derived intermolecular-contact descriptors, the present electrostatic-embedding approach provides a complementary molecular-level description by quantifying how the crystal environment modifies dipole moments, ground-to-excited-state dipole contrast, and β_tot_ of the embedded asymmetric unit.

The comparison with Ashcroft et al.^27^ is also informative, since they used multiphase structural models to show that the hyperpolarizability of DAST is substantially reduced when going from an isolated molecular description to effective crystalline descriptors. They reported β_0_^DFT^ = 159 × 10^−30^ esu for gas-phase DAST, whereas the effective crystalline values were much smaller, with β_0_^MM^ = 17−43 × 10^−30^ esu obtained from multipolar charge-density models. In the present study, DAST also undergoes crystal-induced attenuation, with β_tot_ decreasing from 15,423.58 au in the isolated ion pair to 11,672.54 au in the embedded crystal environment, equivalent to approximately 133.25 and 100.84 × 10^−30^ esu, respectively. Although the reduction predicted here is less pronounced than the effective crystalline attenuation reported by Ashcroft et al., the qualitative trend is the same: the DAST crystal environment does not simply preserve the isolated molecular hyperpolarizability. This difference in magnitude is expected because the present work describes β_tot_ as a local molecular descriptor of the complete asymmetric unit within an electrostatic-embedding model, whereas the crystalline descriptors reported by Ashcroft et al. include additional solid-state effects associated with tensor projection, interionic interactions, and multipolar crystal-lattice contributions.

Overall, these comparisons show that the present results are consistent with the literature in demonstrating that isolated hyperpolarizability alone is not sufficient to predict the nonlinear response of ionic organic crystals. The electrostatic-embedding results further show that the crystal field may attenuate β_*tot*_, as observed for DAST and several 6MNEP-related GUR/GUS crystals; preserve a large response, as observed for DSTMS and DAPSH; or enhance the embedded response, as observed for DSCHS, DSNS-1, and MBST. Therefore, the present data reinforce the need to treat the asymmetric unit explicitly within its crystalline electrostatic environment when comparing local NLO descriptors across structurally diverse ionic salts.

## 4 Conclusion

In summary, the present results show that the local nonlinear response of embedded ion pairs in noncentrosymmetric ionic organic crystals cannot be inferred from crystal symmetry or isolated molecular hyperpolarizability alone. Across the *P*1, *Pn*, *P*2_1_2_1_2_1_, and *Cc* salts examined here, the calculations indicate that the crystal environment can substantially modify both the dipole moment and the static first hyperpolarizability of the asymmetric unit. The relevant crystal field effect is better understood as a local electrostatic perturbation of the embedded ion pair rather than as a simple consequence of global packing density or crystallographic symmetry.

Within the present electrostatic-embedding framework, two broad response patterns emerge. Classical stilbazolium-based salts generally retain comparatively large embedded hyperpolarizabilities, with DAPSH, DSCHS, DSNS-1, and DSTMS remaining among the most responsive systems in the series. In contrast, most 6MNEP-related GUR/GUS crystals exhibit stronger attenuation upon embedding, with the crystal environment often reducing the calculated β_tot_ of the asymmetric unit relative to the isolated ion pair. MBST behaves as a structurally related but distinct case: although it belongs to the broader group of methoxy-substituted push−pull salts, its embedded β_tot_ is slightly enhanced relative to the isolated value. Similarly, DSCHS and DSNS-1 also show crystal-induced enhancement, indicating that selected local packing arrangements and electrostatic environments can reinforce, rather than suppress, the molecular response.

The comparison between β_tot_ and β_tot_^TL^ further shows that the dominant low-energy transition captures part of the qualitative hierarchy of the series, but not always the full quantitative response. Systems with favorable excitation energies, large transition dipole moments, significant vectorial Δμ, and near-collinear ground- and excited-state dipole vectors tend to display larger two-level estimates. However, discrepancies between β_tot_ and β_tot_^TL^ demonstrate that multistate contributions, tensor-component cancellation, and crystal field-induced redistribution of the response remain relevant in several cases. Thus, β_tot_^TL^ should be regarded as a mechanistic and semiquantitative descriptor, whereas the full DFT β_tot_ remains necessary to describe the complete embedded static first hyperpolarizability.

Taken together, these findings show that large isolated β_tot_ values or pronounced charge-transfer character do not by themselves guarantee a larger embedded molecular response in the crystal environment. The crystal field can attenuate, preserve, or enhance the local nonlinear response depending on ion-pair composition, local packing arrangement, and the orientation of the molecular response within the surrounding electrostatic field. The results therefore support the use of explicit crystal embedding as a comparative framework for understanding how lattice organization and ion-pair identity modulate local dipolar and hyperpolarizability descriptors in acentric ionic organic crystals.

## Supplementary Material



## Notes

Additional data supporting the electrostatic-embedding analysis; and convergence profiles of the self-consistent embedding procedure for the dipole moments of the asymmetric units, crystallographic data, and global packing descriptors for all investigated salts, functional-dependent ground- and excited-state dipole moments, and TD-DFT parameters associated with the crucial electronic transitions used in the two-level model (PDF)
