# Supplementary material for: Crystal-Field Effects on Dipole Moments and Static First Hyperpolarizability in Noncentrosymmetric Ionic Organic Crystals
Source: ACS Omega. 2026 Jul 16;11(29):44413–24. doi: 10.1021/acsomega.6c05167 (PMC13425316; doi:10.1021/acsomega.6c05167)
Supplement: Supplementary file 1 [file ao6c05167_si_001.pdf]

# Supporting Information

## Crystal-Field Effects on Dipole Moments and Static First Hyperpolarizability in Non-Centrosymmetric Ionic Organic Crystals

Salviano A. Leão, Augusto César de Jesus, Marcos A. Castro and Tertius L. Fonseca*
Instituto de Física, Universidade Federal de Goiás, Goiânia-GO, 74690-900, Brazil.

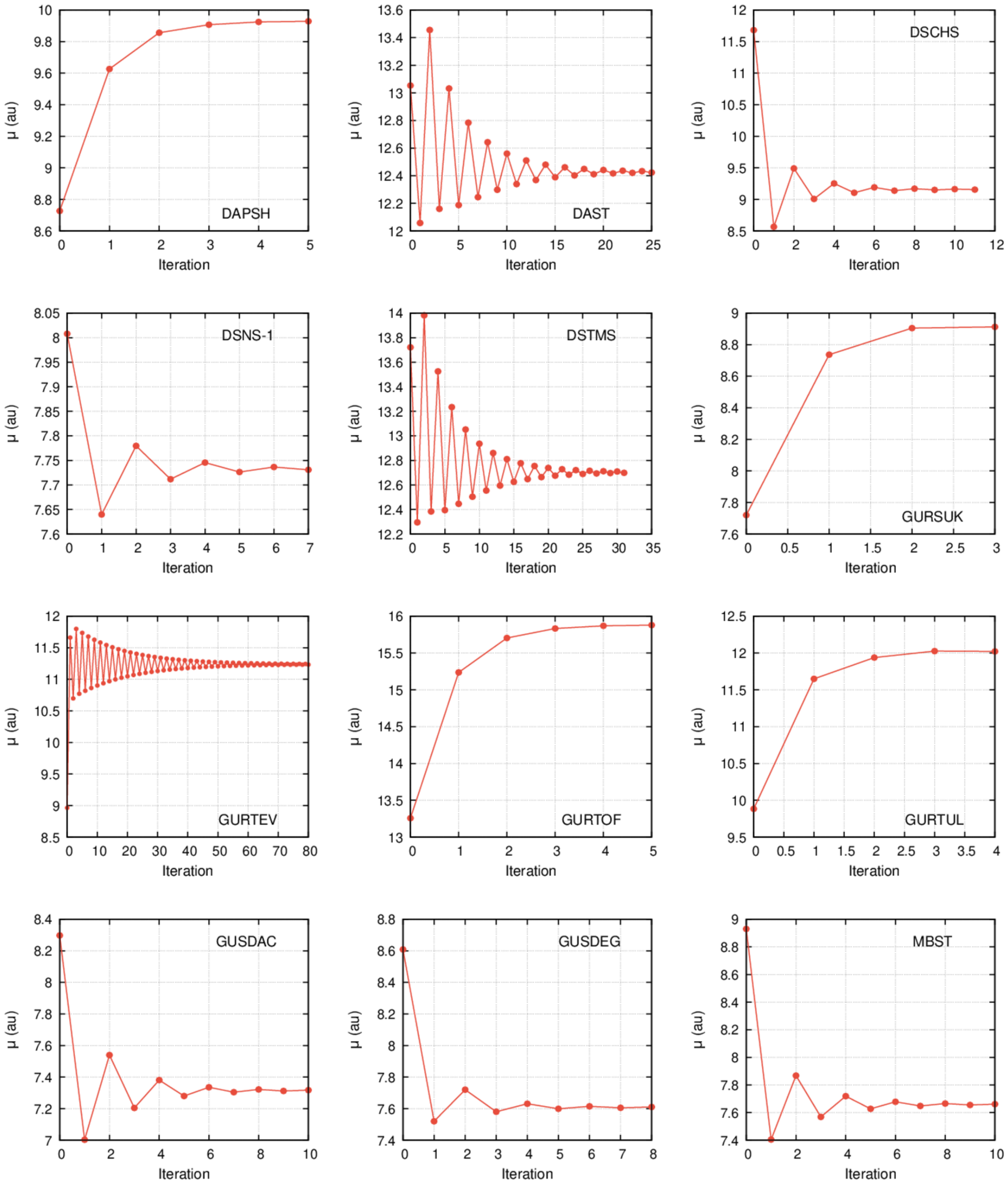


**Figure S1.** Convergence of the calculated dipole moment of the asymmetric units of the non-centrosymmetric ionic crystals analyzed in this work as a function of the iteration step in the self-consistent electrostatic-embedding procedure. The curves illustrate the stabilization of the local dipolar response of the embedded asymmetric units within the adopted embedding model.

**Table S1.** Crystal data, space-group symmetry, and crystallographic descriptors of global packing compactness for the investigated salts: Z, number of asymmetric units per unit cell; V/Z, volume per asymmetric unit; and ρ, crystal density.

| Refcode | System | Space group | Z | V (Å$^3$) | V/Z (Å$^3$) | ρ (g cm$^{-3}$) |
|---|---|---|---|---|---|---|
| DAPSH<br>CCDC 163560 | Monoclinic | Cc | 4 | 1967.30 | 491.80 | 1.507 |
| DAST<br>CCDC 1175744 | Monoclinic | Cc | 4 | 2098.18 | 524.55 | 1.300 |
| DSCHS<br>CCDC 781566 | Triclinic | P1 | 1 | 524.40 | 524.40 | 1.446 |
| DSNS-1<br>CCDC 292763 | Triclinic | P1 | 1 | 558.92 | 558.92 | 1.327 |
| DSTMS<br>CCDC 277597 | Monoclinic | Cc | 4 | 2261.20 | 565.30 | 1.288 |
| GURTOF<br>CCDC 1960849 | Monoclinic | Pn | 2 | 1116.27 | 558.13 | 1.492 |
| GURSUK<br>CCDC 1960840 | Orthorhombic | $P2_12_12_1$ | 4 | 2158.36 | 539.59 | 1.442 |
| GURTEV<br>CCDC 1960847 | Orthorhombic | $P2_12_12_1$ | 4 | 2144.25 | 536.06 | 1.419 |
| GURTUL<br>CCDC 1961452 | Orthorhombic | $P2_12_12_1$ | 4 | 2103.78 | 525.95 | 1.435 |
| GUSDAC<br>CCDC 1867834 | Triclinic | P1 | 1 | 539.49 | 539.49 | 1.377 |
| GUSDEG<br>CCDC 1867835 | Triclinic | P1 | 1 | 539.55 | 539.55 | 1.473 |
| MBST<br>CCDC 774844 | Triclinic | P1 | 1 | 495.52 | 495.53 | 1.332 |

**Table S2.** DFT/6-311+G(d) results for the ground-state dipole moment ($\mu_g$, in a.u.) and TD-DFT/6-311+G(d) results for the excited-state dipole moment ($\mu_e$, in a.u.) of the in-crystal asymmetric units of the ionic crystals.

| Asymmetric Unit | CAM-B3LYP | | LC-BLYP | | M05-2X | | M06-2X | |
|---|---|---|---|---|---|---|---|---|
| | $\mu_g$ | $\mu_e$ | $\mu_g$ | $\mu_e$ | $\mu_g$ | $\mu_e$ | $\mu_g$ | $\mu_e$ |
| DAPSH | 9.93 | 10.12 | 9.48 | 9.99 | 9.50 | 10.07 | 9.49 | 9.99 |
| DAST | 12.42 | 12.44 | 12.61 | 12.43 | 12.45 | 12.48 | 12.41 | 12.45 |
| DSCHS | 9.15 | 8.32 | 9.57 | 8.56 | 9.19 | 7.75 | 9.15 | 8.02 |
| DSNS-1 | 7.73 | 7.88 | 7.96 | 7.99 | 7.75 | 7.90 | 7.73 | 7.88 |
| DSTMS | 12.70 | 12.69 | 13.01 | 12.65 | 12.71 | 12.71 | 12.67 | 12.68 |
| GURSUK | 8.91 | 11.63 | 8.83 | 11.12 | 8.91 | 11.73 | 8.88 | 11.77 |
| GURTEV | 11.23 | 9.15 | 11.35 | 9.48 | 11.26 | 9.12 | 11.30 | 9.06 |
| GURTOF | 15.88 | 13.68 | 16.05 | 14.26 | 15.90 | 13.73 | 15.90 | 13.56 |
| GURTUL | 12.02 | 12.42 | 12.13 | 11.17 | 12.03 | 11.54 | 12.04 | 11.13 |
| GUSDAC | 7.32 | 8.11 | 7.35 | 8.20 | 7.34 | 8.16 | 7.30 | 8.47 |
| GUSDEG | 7.61 | 4.33 | 7.69 | 8.16 | 7.64 | 8.35 | 7.63 | 8.31 |
| MBST | 7.66 | 7.77 | 7.79 | 7.77 | 7.70 | 7.79 | 7.68 | 7.76 |

**Table S3.** CAM-B3LYP results for the excitation energy ($E_{ge}$, in eV) and oscillator strength (f) of the crucial electronic transition, the ground- and excited-state dipole moment difference ($\Delta\mu = |\vec{\mu}_e - \vec{\mu}_g|$, in a.u.), transition dipole moment ($\mu_t$, in a.u.), cosine of the angle between the dipole moment vectors of the ground state and the excited state (cosθ) and the first hyperpolarizability of the two-level model [($\beta_{tot}^{TL} \propto (\mu_{tr}^2\Delta\mu/E_{ge}^2)\sqrt{1+8\cos^2\theta}$), in a. u.] of in-crystal asymmetric units.

| Asymmetric Unit | f | $E_{ge}$ | $\mu_{tr}$ | $\Delta\mu$ | cosθ | $\beta_{tot}^{TL}$ |
|---|---|---|---|---|---|---|
| DAPSH | 1.4901 | 2.6322 | 4.4293 | 3.1834 | -0.9998 | 17495.09 |
| DAST | 1.1549 | 2.3776 | 4.8312 | 0.7508 | -0.9815 | 8127.56 |
| DSCHS | 1.1126 | 2.5345 | 4.3782 | 1.9981 | -0.9860 | 15695.03 |
| DSNS-1 | 0.8449 | 2.7282 | 3.4087 | 4.4856 | -0.9977 | 15738.55 |
| DSTMS | 0.7437 | 2.0920 | 4.6658 | 0.6929 | -0.9714 | 8953.30 |
| GURSUK | 1.3098 | 3.4136 | 4.0959 | 3.9730 | 0.9981 | 15220.56 |
| GURTEV | 1.3140 | 3.5366 | 4.1048 | 3.4105 | 0.9975 | 12218.90 |
| GURTOF | 1.0290 | 3.6593 | 3.6675 | 3.1599 | -0.9947 | 8420.32 |
| GURTUL | 1.2040 | 3.3756 | 3.6009 | 3.1071 | -0.9389 | 8914.13 |
| GUSDAC | 1.0934 | 3.8781 | 3.4842 | 2.0570 | -0.9919 | 4393.80 |
| GUSDEG | 1.0166 | 3.4973 | 2.8182 | 3.5129 | -0.1324 | 2164.11 |
| MBST | 0.9404 | 3.2098 | 3.4027 | 3.5143 | -0.9998 | 10525.20 |

**Table S4.** LC-B3LYP results for the excitation energy ($E_{ge}$, in eV) and oscillator strength (f) of the crucial electronic transition, the ground- and excited-state dipole moment difference ($\Delta\mu = | \vec{\mu}_e - \vec{\mu}_g |$, in a.u.), transition dipole moment ($\mu_t$, in a.u.), cosine of the angle between the dipole moment vectors of the ground state and the excited state (cosθ) and the first hyperpolarizability of the two-level model [($\beta_{tot}^{TL} \propto (\mu_{tr}^2 \Delta\mu / E_{ge}^2)\sqrt{1 + 8\cos^2\theta}$), in a. u.] of in-crystal asymmetric units.

| Asymmetric Unit | f | $E_{ge}$ | $\mu_{tr}$ | $\Delta\mu$ | cosθ | $\beta_{tot}^{TL}$ |
|---|---|---|---|---|---|---|
| DAPSH | 1.5501 | 3.1911 | 4.4527 | 3.4848 | 0.9999 | 18083.13 |
| DAST | 1.5566 | 2.4317 | 5.1116 | 1.1649 | -0.9999 | 13718.60 |
| DSCHS | 1.3501 | 2.7437 | 4.4817 | 3.4585 | -0.9990 | 24572.79 |
| DSNS-1 | 1.0294 | 3.2936 | 3.5717 | 5.3514 | -0.9997 | 16768.92 |
| DSTMS | 1.2398 | 2.1974 | 4.7990 | 2.2848 | -0.9993 | 29029.81 |
| GURSUK | 1.5850 | 3.8014 | 4.1254 | 3.5498 | -0.9967 | 11110.78 |
| GURTEV | 1.5599 | 3.9052 | 4.0378 | 2.9495 | 0.9968 | 8380.75 |
| GURTOF | 1.2717 | 3.9848 | 3.6091 | 2.5414 | -0.9937 | 5525.56 |
| GURTUL | 1.3916 | 3.7552 | 3.8893 | 3.1937 | -0.9957 | 9096.61 |
| GUSDAC | 1.2063 | 4.1687 | 3.4368 | 1.7561 | -0.9980 | 3175.62 |
| GUSDEG | 1.2148 | 3.8856 | 3.5722 | 3.0408 | -0.9905 | 6792.52 |
| MBST | 1.0156 | 3.5767 | 3.4045 | 3.5998 | -0.9999 | 8692.57 |

**Table S5.** M05-2X results for the excitation energy ($E_{ge}$, in eV) and oscillator strength (f) of the crucial electronic transition, the ground- and excited-state dipole moment difference ($\Delta\mu = |\vec{\mu}_e - \vec{\mu}_g|$, in a.u.), transition dipole moment ($\mu_t$, in a.u.), cosine of the angle between the dipole moment vectors of the ground state and the excited state (cosθ) and the first hyperpolarizability of the two-level model [($\beta_{tot}^{TL} \propto (\mu_{tr}^2 \Delta\mu / E_{ge}^2)\sqrt{1 + 8\cos^2\theta}$), in a. u.] of in-crystal asymmetric units.

| Asymmetric Unit | f | $E_{ge}$ | $\mu_{tr}$ | $\Delta\mu$ | cosθ | $\beta_{tot}^{TL}$ |
|---|---|---|---|---|---|---|
| DAPSH | 1.4965 | 2.9752 | 4.5311 | 3.0991 | 0.9998 | 19155.50 |
| DAST | 1.3672 | 2.4015 | 4.8206 | 0.6786 | -0.9745 | 7123.20 |
| DSCHS | 1.1312 | 2.5376 | 4.2656 | 2.0259 | -0.8678 | 13480.11 |
| DSNS-1 | 0.8686 | 2.7491 | 3.5913 | 4.6927 | 0.9995 | 21335.17 |
| DSTMS | 1.1346 | 2.1203 | 4.6735 | 0.6070 | -0.9603 | 7583.96 |
| GURSUK | 1.3942 | 3.4316 | 4.0722 | 4.1057 | 0.9983 | 15386.84 |
| GURTEV | 1.464 | 3.5659 | 4.0936 | 3.5078 | 0.9977 | 12296.46 |
| GURTOF | 1.2248 | 3.6889 | 3.6812 | 3.1250 | -0.9946 | 8254.62 |
| GURTUL | 1.2271 | 3.4015 | 3.8373 | 3.2919 | -0.9979 | 11144.96 |
| GUSDAC | 1.1835 | 3.9061 | 3.5167 | 2.0429 | -0.9943 | 4391.28 |
| GUSDEG | 1.1079 | 3.5453 | 3.5714 | 3.7302 | 0.9905 | 10003.76 |
| MBST | 0.9299 | 3.2596 | 3.4124 | 3.4607 | -0.9999 | 10108.49 |

**Table S6.** M06-2X results for the excitation energy ($E_{ge}$, in eV) and oscillator strength (f) of the crucial electronic transition, the ground- and excited-state dipole moment difference ($\Delta\mu = |\vec{\mu}_e - \vec{\mu}_g|$, in a.u.), transition dipole moment ($\mu_t$, in a.u.), cosine of the angle between the dipole moment vectors of the ground state and the excited state (cosθ) and the first hyperpolarizability of the two-level model [($\beta_{tot}^{TL} \propto (\mu_{tr}^2 \Delta\mu / E_{ge}^2)\sqrt{1 + 8\cos^2\theta}$), in a. u.] of in-crystal asymmetric units.

| Asymmetric Unit | f | $E_{ge}$ | $\mu_{tr}$ | $\Delta\mu$ | cosθ | $\beta_{tot}^{TL}$ |
|---|---|---|---|---|---|---|
| DAPSH | 1.4343 | 2.9127 | 4.4833 | 3.1353 | 0.9999 | 19798.38 |
| DAST | 1.3420 | 2.3678 | 4.8098 | 0.6318 | -0.9683 | 6753.56 |
| DSCHS | 1.1465 | 2.5028 | 4.3241 | 1.8209 | -0.9259 | 13536.69 |
| DSNS-1 | 0.8362 | 2.6638 | 3.5794 | 4.6691 | -0.9994 | 22457.64 |
| DSTMS | 1.1130 | 2.0878 | 4.6648 | 0.5457 | -0.9488 | 6931.86 |
| GURSUK | 1.3743 | 3.3943 | 4.0653 | 4.2578 | 0.9985 | 16257.19 |
| GURTEV | 1.4282 | 3.5353 | 4.0607 | 3.6683 | 0.9980 | 12876.26 |
| GURTOF | 1.1960 | 3.6588 | 3.6528 | 3.3860 | -0.9953 | 8958.01 |
| GURTUL | 1.2374 | 3.3627 | 3.8755 | 3.7799 | -0.9993 | 13373.86 |
| GUSDAC | 1.1951 | 3.8826 | 3.5445 | 2.3804 | -0.9985 | 5280.90 |
| GUSDEG | 1.0613 | 3.5006 | 3.5178 | 3.9183 | -0.9874 | 10428.53 |
| MBST | 0.8925 | 3.1818 | 3.3837 | 3.5848 | 0.9998 | 10804.24 |
